# Relationship between Capillary Refill Time at Triage and Abnormal Clinical Condition: A Prospective Study

**DOI:** 10.2174/1874434601711010084

**Published:** 2017-07-26

**Authors:** Claudia M. Sansone, Fabiano Prendin, Greta Giordano, Paola Casati, Anne Destrebecq, Stefano Terzoni

**Affiliations:** 1 Nurse, Azienda Ospedaliero-Universitaria Maggiore della Carità di Novara, Corso Mazzini 18, 28100 Novara, Italy; 2 Nurse, Emergency Department, San Paolo teaching hospital, Via A. di Rudinì, 8 – 20142 Milan, Italy; 3 Nurse, ASST Santi Paolo e Carlo, presidio San Paolo, Via A. di Rudinì, 8 – 20142 Milan, Italy; 4 Operating theatre, San Raffaele Hospital, Via Olgettina 60, 20132 Milan, Italy; 5 Associate professor of Nursing, University of Milan, Via Ovada, 26 – 20142 Milan, Italy; 6 PhD, Tutor Nurse, San Paolo bachelor school of Nursing, San Paolo teaching hospital, Via Ovada, 26 – 20142 Milan, Italy

**Keywords:** Assessment, Capillary refill time, Clinical conditions, Nursing, Perfusion, Triage

## Abstract

**Background:**

Capillary refill time has been studied in literature as a perfusion indicator. Two pilot studies have proposed possible reference values in healthy adults. No data exist regarding capillary refill time as an indicator of abnormal clinical conditions in adults, which might be of help for triage nurses.

**Objective:**

We wanted to assess if any relationships existed, between altered capillary refill time and abnormal clinical conditions in the emergency department. We investigated relations between capillary refill time and vital signs recorded in triage and blood tests, by analyzing the clinical records. Mortality at 24 hours, 7 days and over 14 days was investigated by calling the patients after discharge.

**Method:**

Observational, single-center study on a sample of consecutive patients aged ≥ 18 years in the Emergency Department of a major Milan hospital, from June to October 2014. Multivariate logistic regression was used to investigate the impact of clinical variables on capillary refill time.

**Results:**

1001 patients were enrolled, aged 59 ± 21 (473 aged 65 or more). Longer refill times were found in patients admitted to hospital units after medical consultations in the emergency department compared to those discharged or sent to outpatients. In elderly patients, statistically significant association was found between increased capillary refill time and sepsis (sensitivity 100%, specificity 83.33%, area under the receiver operating characteristics curve 65.95% CI 47-83), oxygen saturation, mean blood pressure, and lactates. In persons aged 45 to 64, altered refill times were associated with abnormal values of glicemia, platelets, and urea.

**Conclusion:**

Capillary refill time can be used by nurses at triage as a complementary parameter to normal vital signs. This is one of the few studies investigating refill time in adult patients.

## 1 INTRODUCTION

Capillary refill time (CRT) is defined as “the time needed by a distal body region (e.g. a fingertip) to regain the original color after having been compressed” [1, 2]. Some authors [3-6] have proposed CRT as an indicator of perfusion in adults: in case of hypovolemic shock, peripheral vasoconstriction is a fundamental protection mechanism, aimed at saving blood for vital organs. Also, a rationale exists for investigating this parameter in patients with medical conditions related to inadequate perfusion, e.g. sepsis. The current literature provides very few information about pathologic situations capable of modifying CRT. Based on the available studies, all international algorithms of prehospital trauma care include CRT assessment: the European Resuscitation Council includes CRT as part of the assessment that should be conducted regarding circulation in the ABCDE algorithm (Airways, Breathing, Circulation, Disability, Exposure) [7]. Some indications appear in pediatric studies, as capillary refill time is mentioned in the Surviving Sepsis Campaign section pediatric patients. The guidelines recommend to obtain a capillary refill time of 2 seconds or less as an initial therapeutic endpoint of resuscitation in septic shock [8]. Mrgan, *et al.* [9] found a statistically significant association between abnormal CRT (as defined by Schrigger and Barraf) and mortality after 24 hours (OR=5.81, 95%CI=[1.36;24.79], p<.001) and 7 days after discharge from the emergency department (OR=4.24, 95%CI=[1.88;9.56], p<.001) although the positive predictive value of 24-hour mortality was as low as 15%. However, the authors provide no information regarding other abnormal conditions possibly related to alterations of CRT.

As regards assessment of CRT, Champion [10] suggested a maximum refill time of 2 seconds, while Schrigger and Barraf [6] differentiated the cutoff values, based on age and gender, in healthy patients. The refill times they identified as normal do not exceed 1.9 seconds for males and 2.9 for females aged 18 to 64, and 1.8 seconds in both genders aged 65 or more. All the abovementioned values have been studied under controlled conditions of temperature (21°C). Considering the practical difficulties related to decimal values, the authors have also suggested to consider as physiological a refill time of 2 and 3 seconds under 65 years of age, for males and females respectively, as well as 4 seconds in both genders for elderly persons [1].

Recently, CRT at triage has been suggested as a parameter of blood perfusion on small samples of adults, without drawing firm conclusions. Therefore, data regarding possible association between CRT and abnormal clinical condition in adults are lacking. These data could be of interest for triage nurses: should relations with specific conditions emerge, CRT might be used as a useful indicator for assessing the degree of urgency of patients. Finally, considering the unsatisfactory predictive value of mortality currently available, as well as the lack of a systematic investigation on large samples in this field, the possibility of consolidating this information with detailed analyses and longer follow-up of patients appears to be of interest.

The study presented in this paper aims to investigate possible relations between alterations of CRT in adult patients admitted to an Emergency Department (ED), and abnormal clinical conditions, as well as mortality after 24 hours, 1 week, and two weeks after discharge from the ED.

## 2 MATERIALS AND METHODOLOGY

A prospective, observational, single center study was conducted. We recruited a non-randomized sample of consecutive patients, aged 18 or more, who presented to the emergency department of the San Paolo teaching hospital in Milan, Italy, between June 1 and October 20, 2014. All patients with codes of emergency or urgency were included. Pediatric patients, newborns, and non-urgent cases were excluded. During the triage process in the ER, capillary refill time was assessed in all patients, in the same conditions of temperature (air conditioning room, 21°C temperature as described in the literature) and with the same clock [9-19] to avoid any potential bias. The criteria for obtaining the refill time data included compressing the fingertip of the second finger (left or right hand was irrelevant, according to the literature [1]), releasing, and measuring the time required to regain the initial color. A visual analogic scale (VAS) was used for measuring pain; all medical diagnoses, as well as the results of all blood tests, were recorded. Standard reference ranges were used for all tests and vital signs, according to the literature [11, 12]. Several cutoff values were taken into consideration for this study. At first, all analyses were performed according to the criteria defined by Schrigger and Barraf [1], thus accepting 2 seconds as a cutoff for all patients. Considering that most of the available literature refers to healthy patients, while those in pathologic conditions could have longer refill times, the analysis was repeated after setting the cutoff to 3 seconds for all patients, and 4 seconds for elder persons respectively. We chose to avoid decimal cutoffs, since this would generate potential biases during the measurement: correct assessment of the refill time might actually require multiple attempts in order to obtain reliable data, thus requiring repeated pressure of the capillary bed of the fingertip, which can be misleading. A capillary refill time of 2 seconds was choses as a cutoff. All analyses were repeated considering 3 seconds and 4 seconds as alternative cutoff values. Differences between CRT values in patients with different priority codes at nursing triage were described. Mortality was assessed through telephone contact after 24 hours, 1 week, and 2 weeks from discharge. In case the patients were transferred to other hospital units, mortality was assessed through their medical records. A written form with the same questions for all patients was used in case of telephone contact.

## 2.1 Statistical Analysis

Wilcoxon’s signed-rank test was used for comparing median values (*e.g*. median capillary refill times). The values of all blood tests and vital parameters were dychotomized (altered/non-altered) according to the abovementioned criteria, and chi-square tests for association were performed, in order to assess possible relations with capillary refill time. Since patients are complex systems, and many variables are related to each other, a multivariate stepwise logistic regression model was used, to assess the role of all clinical indicators in the overall presentation of our patients. In such model, alterations of CRT were studied in relationship with vital signs and blood tests. Finally, a sensitivity and specificity analysis was conducted, to determine whether CRT values could help distinguishing generic infections from sepsis. In this analysis, a ROC curve (Receiver Operating Characteristics) was used. 95% confidence intervals were calculated in all inferential analyses; the results were considered statistically significant in presence of p-values below .05. For each analysis, the actual number of patients was taken into account (see Table **2**) as not all patients underwent the same tests. All data were collected through the FirstAid software, officially in use in the Emergency Department, and analyzed with STATA® 11 for Windows. We complied with the rules of the local Ethical committee; the hospital management approved the study, which was conducted in respect of the principles stated in the Declaration of Helsinki [20]. Patients’ data were treated anonymously, as all subjects were identified by the unique code provided by the hospital software. All data were treated according to the national Italian law on privacy protection.

## 3 RESULTS

1001 consecutive patients were enrolled (482 males, 48.15%, and 519 females, 51.85%), aged 59 ± 21 (473 aged 65 or more). Table **1** summarizes the categories of medical diagnoses of the patients.

As a first finding, significantly higher refill times were found in patients admitted to hospital units after medical consultations in the ER (n=566, Me=4, IQR= [3, 5]. 19 refused to be admitted, 1 was transferred to another hospital) if compared to those discharged (n=221, Me=3, IQR= [2, 4]) or sent to outpatients for further medical activities (n=194, Me=3, IQR= [2, 4]), p<.001 for all comparisons. 413 patients (41.25%) had capillary refill times above 2 seconds. Of these, 12 had the highest priority code, 214 had intermediate priority codes and 187 had low priority. Of note, the number of patients aged 65 or more increased with the severity of clinical conditions, if compared to the number of younger persons (65 *vs* 122 among low-priority codes, 133 *vs* 81 among intermediate priority codes, and 10 *vs* 2 among high priority patients). Statistically significant associations were found between single variables and altered refill times. Considering that some parameters can be altered mostly in the elderly, or at least in the middle-aged, we chose to divide patients into three separate ranges, based on their age (18-44, 45-64, and 65 or more), as defined in the literature [11].

Table **2** shows the results of the analysis and reports the number of patients for each blood parameter, since not all patients underwent the same exams. Values are presented as odds ratios and 95% confidence intervals. Statistically significant results are marked in bold. Only significant p-values are reported: in all other cases, the analysis reported p-values above 0.05.

Statistically significant associations were found between altered capillary refill time and diastolic hypertension in younger patients (aged 18-44). In middle-aged persons (45-64), CRT was associated with hyperglycemia, thrombocytopenia, and high levels of urea and alanine transaminase.

A clinically relevant finding emerged in elderly patients, in which CRT alterations showed statistically significant and strong associations with blood oxygen saturation <90%, diastolic hypotension, mean blood pressure, and lactates. According to the literature, such parameters can be found in patients with sepsis. In the multivariate logistic regression model, the associations were confirmed, as shown in Table **3**.

Actually, only seven patients were diagnosed with sepsis in our sample, all aged 65 or more. All seven had capillary refill time alterations; based on this finding, we decided to further investigate this aspect, and we conducted a sensitivity analysis to determine whether CRT could be able to distinguish between generic infections and sepsis. Sensitivity was 100%, as CRT was elevated in all septic patients; specificity was 83.33%, with an area under the ROC (Receiver Operating Characteristics) curve of .65, 95%CI=[.47-.83]. Although not fully satisfactory, these latter findings account for further data collection and investigation in future research, especially considering the interesting data regarding specificity.

## 3.2 Mortality

We were able to retrieve the data of all 1001 patients, through telephone contact or medical records. 25 patients enrolled for this study died, out of 1001 (2.49%). They had been admitted to the emergency department for trauma (n=7), sepsis (n=3), hemorrhage (n=3), central nervous system disorders (n=3), and respiratory (n=2), gastroenteric (n=2), nephrological (n=1) or cardiological (n=1) conditions. The remaining 3 had generic diagnoses, which required further medical assessment (*e.g*. “acute pain”).

Out of those 25, 4 died in 24 hours, 2 in a week, and 19 after two weeks or more. We sought to find associations between capillary refill time alterations and mortality; Table **4** summarizes the results.

No statistically significant relationship was found between altered CRT values and mortality, regardless of age.

## 4 DISCUSSION

The findings of the present study show an association between prolonged CRT in elder adults and several clinical indicators of septic conditions. Such relationships appear to be strong, as confirmed by odds ratios, and stay evident in both bivariate and multivariate analysis. Sensitivity analysis yielded clear distinction between patients diagnosed with sepsis and persons with generic infections (*e.g*. urinary tract infections). These findings appear to be the first in literature, and deserve further investigation. In persons aged 45 to 64, prolonged CRT was associated with hyperglycemia, as well as alterations in the amount of alanine transaminase and urea; these two latter findings might be of interest and need to be investigated in-depth. As to our knowledge, no papers in the existing literature have investigated this relationship in adults so far. In particular, hyperglycemia has only been investigated in infants [21]. In patients aged 18 to 44, an association was found between CRT and diastolic hypertension. Overall, from the point of view of pathophysiology, the results of this investigation appear to be congruent with the characteristics and age ranges of diseases onset. The small number of patients diagnosed with sepsis prevents from drawing firm conclusions; however, the association of indicators of sepsis with prolonged CRT strongly suggest that refill time can actually be studied as an indicator of sepsis in the general elder population. Some authors, such as Brunauer *et al.* [22] have recently suggested that CRT is associated with pulsatility index of the liver and intestines; pulsatility index is a sonographic surrogate of vascular tone, meant to be assessed in visceral organs in patients with early septic shock. Our findings support such results and point out the importance of assessing CRT in patients with suspected sepsis, as suggested by Postelnicu *et al.* [23].

As regards mortality, in this sample of patients no significant associations with CRT were found. Other authors have studied CRT in different populations, finding a relationship between refill time and 14-day mortality after resuscitation in patients with septic shock [24]. However, no studies appear to be available in literature regarding patients with our inclusion criteria.

## 5 LIMITATIONS

This was a single-center, non-randomized study, conducted in a general hospital. The number of patients with sepsis only allows preliminary conclusions on the real capacity of CRT to distinguish between generic infections and sepsis. Further investigations are needed to strengthen this part of the study. In our sample, many different types of problems were diagnosed; therefore, not all patients underwent the same blood tests. This limitation is partially amended by the overall large number of persons we studied, which allows drawing preliminary conclusions from our data.

## CONCLUSION

Capillary refill time has shown association with several conditions, which change over the lifespan. Older patients have a broader range of abnormal condition, which present parallel to CRT alterations.

The potential utility of CRT as a clinical indicator is also supported by the fact that the highest CRT values were found in the patients admitted to hospital units after consultation in the ED. This suggests that CRT alteration become more evident in case of severe and urgent problems.

This is one of the few studied to have investigated the potential role of CRT at triage as a clinical indicator in adults at triage. Other authors have studied the same topic in pediatric patients, but data regarding adult patients were lacking. The present investigation has identified several categories of health problems; in the future, it would be useful to understand if CRT can improve sensitivity and specificity of the current diagnostic methods for such conditions. If so, CRT would become a very interesting indicator for both triage nurses and physicians in the emergency department. Further investigation on large samples of patients with specific diseases is needed to compare the accuracy of diagnoses made with or without taking capillary refill time into account.

## Figures and Tables

**Table 1:** Medical diagnoses.

**Condition**	**Freq.**	**%**
Unspecified illness	14	1,40
Alterated body temperature	14	1,40
Infections/sepsis	104	10,39
Circulatory	106	10,59
Haemorrage	67	6,69
Respiratory	39	3,90
Neurological	76	7,59
Urological	42	4,20
Gastroenteric	130	12,99
Metabolis	25	2,50
Pain	52	5,19
Trauma	267	26,67
Psychiatric/other	65	6,49
**Total**	**1001**	**100**

**Table 2:** Associations between altered refill times and blood exams.

**Blood parameter**	**No. patients**	**Aged 18-44**	**Aged 45-64**	**Aged 65 or more**
Oxygen saturation	1001	NA*	1.30[.01-103.31]	2.48[.96-6.90], p=.03
Heart rate	1001	1.15[.64-2.05]	.72[.37-1.37]	1.16[.74-1.81]
Respiratory rate	98	.66[.21-2.18]	.98[.31-1.37]	1.03[.50-2.18]
Hyperglicemia	536	.93[.44-1.94]	2.08[1.01-4.63],p=.04	1.33[.77-2.30]
Systolic pressure	1001	1.39[.66-2.88]	1.03[.57-1.84]	.84[.57-1.23]
Dyastolic pressure	1001	2.37[1.01-5.67],p=.02	.81[.40-1.63]	2.0[1.08-3.74],p=.01
Red cells count	586	.77[.39-1.49]	.95[.45-2.01]	118[.76-1.82]
White cells count	586	1.74[.86-3.50	1.04[.48-2.26]	.96[.57-1.62]
Haemoglobin	586	1.21[.60-2.40]	.94[.44-1.99]	.91[.54-1.53]
Hematocrit	586	1.15[.51-2.56]	1.07[.44-1.99]	.82[.45-1.47]
Mean cell volume	586	1.53[.63-3.60]	.46[.15-1.33]	1.25[.67-2.31]
Platelets	586	1.51[.54-4.09]	4.77[1.16-27.6],p=.01	1.36[.59-3.16]
C-reactive protein	479	1.08[.52-2.22]	1.30[.55-3.05]	.70[.40-1.24]
Urea	271	1.37[.47-3.89]	9.2[1.01-10.72].p=.03	1.00[-45-2.21]
Creatinin	575	.66[.29-1.43]	1.18[.53-2.61]	.94[.53-1.69]
Aspartate-aminotransferase	516	1.00[.24-3.69]	3.22[.82-15.08]	1.41[.55-3.64]
Alanine transaminase	516	1.30[.30-5.07]	3.46[1.02-16.06],p=.03	1.11[.45-2.70]
Sodium	562	.60[.13-2.15]	1.78[.62-5.16]	.98[.46-2.05]
Potassium	554	.86[.40-1.83]	.95[.44-2.04]	1.33[.77-2.30]
Calcium	168	1.00[.18-4.72]	3.8[.16-235.70]	.80[.23-2.61]
Partial Thromboplastine time	256	.50[.01-5.50]	.56[.01-11.56]	.61[.12-2.57]
Troponin	114	1.90[.35-11.31]	1.28[.16-9.73]	.70[.20-2.45]
Lipase	145	.45[.01-6.29]	NA*	2.46[.12-149.37]
Bilirubin	261	.51[.12-1.75]	1.00[.24-4.12]	1.06[.44-2.51]
Albumin	33	.33[.01-52.12]	NA*	1.66[.21-14.85]
pO_2_	95	1.20[.06-22.28]	2.70[.38-19.77]	.60[.38-5.65]
pCO_2_	95	2.25[.16-37.19]	1.8[.25-12.62]	1.31[.36-4.79]
pH	95	4.00[.30-64.00]	1.29[.17-11.24]	1.50[.38-5.65]
Bicarbonates	87	NA*	1.6[.16-21.62]	.87[.7-5.98]
Lactates	80	.65[.13-3.38]	.64[.17-2.31]	3.50[1.25-12.09],p=.008
Mean blood pressure	1001	.01[.001-1.42]	1.98[.22-24.11]	2.63[.89-8.68],p=.04
*NA=Not Applicable, the odds ratio cannot be calculated due to the presence of zeros in the contingency table. The results are presented as OR[95% CI], p-value.				

**Table 3:** Results of the multivariate logistic model.

**Variable**	**OR[95%IC]**	**Standard error**	**p-value**
Oxygen saturation<90%	2.57[1.03-6.45]	1.20	.04
Diastolic hypotension	1.93[1.07-3.45]	.57	.02
Mean blood pressure	2.78[1.00-7.73]	1.45	.04
Lactates	3.75[1.35-10.45]	1.96	.01

**Table 4:** Associations between capillary refill time and mortality.

**Time**	**18-44 years old**	**45-64 years old**	**65+ years old**
24 hours	.00[.00-1.18],p=.46	.00[.00-1.41],p=.24	.00[.00-2.43],p=.20
1 week	.00[.00-2.32],p=.71	.00[.00-1.18],p=.25	1.09[.29-3.85],p=.87
2 weeks or more	.62[.01-7.88],p=.68	.00[.00-1.09],p=.18	.00[.00-.51],p=.37
